# Comparative evaluation of TRIDs : a strategy to improve treatments

**DOI:** 10.1186/s12967-026-08058-5

**Published:** 2026-03-31

**Authors:** Fatima Hariss, Sacha Spelier, Louise Damy, Carmen Sandoval Pacheco, Luca Houpe, Catherine Leroy, Xavier Thuru, Frédéric Becq, Fabrice Lejeune

**Affiliations:** 1 Univ. Lille, Inserm, CHU Lille, CNRS, U1366-UMR9020 – CRCLille – Cancer Research Center of Lille, Lille, F-59000 France; 2 Pediatric Respiratory Medicine, Wilhelmina Children’s Hospital, University Medical Center, Utrecht, 3584 EA The Netherlands; 3 Regenerative Medicine Utrecht, University Medical Center, Utrecht University, Utrecht, 3584 CT The Netherlands; 4 Centre for Living Technologies, University Medical Center, Utrecht University, Utrecht, 3584 CT The Netherlands; 5 Laboratoire de physiopathologie et régulation des transports ioniques, Pôle Biologie Santé, Université de Poitiers, Poitiers, 86073 France

**Keywords:** Nonsense mutation, Translational readthrough, Premature termination codon, Small molecules, Genetic diseases

## Abstract

**Background:**

Translational readthrough represents a promising therapeutic strategy for genetic disorders caused by nonsense mutations. Although multiple translational readthrough-inducing drugs (TRIDs) have been reported, their relative efficacy remains inconsistent across studies, likely due to differences in experimental systems and cellular contexts. A systematic comparison across complementary and clinically relevant models is therefore required to better define their therapeutic potential. This study aims to evaluate and compare the efficiency of different TRIDs used for nonsense mutation correction.

**Methods:**

We performed a comparative evaluation of 12 TRIDs using a panel of cellular models encompassing increasing levels of physiological relevance. These included a firefly luciferase-based reporter assay, human cell lines harboring endogenous TP53 nonsense mutations, patient-derived intestinal organoids carrying CFTR nonsense variants, and Fischer rat thyroid (FRT) cells expressing homozygous CFTR nonsense mutations. Readthrough efficiency was assessed and compared across models.

**Results:**

Across the different systems, 2,6-diaminopurine (DAP), clitocine, SRI-41315, and TLN468 consistently exhibited the highest readthrough activity. However, variations in relative efficacy were observed depending on the assay and cellular context, underscoring the influence of experimental conditions, amino acid incorporation profiles, and cell-specific determinants on readthrough outcomes.

**Conclusions:**

Our findings demonstrate the necessity of evaluating candidate TRIDs across multiple complementary and patient-relevant models to accurately estimate their therapeutic potential. This study identifies several promising compounds for further preclinical development and provides a comparative framework to guide the rational selection of readthrough-inducing agents for translational applications.

**Supplementary information:**

The online version contains supplementary material available at 10.1186/s12967-026-08058-5.

## Background

Genetic diseases are linked to the malfunction of a gene, meaning that multiple types of mutations can lead to the same pathology, with severity depending on the specific type of mutation. One particularly impactful category of variants includes nonsense mutations. A nonsense mutation is a point mutation that converts a codon into a premature termination codon (PTC). The consequence of a PTC is rarely the synthesis of a truncated protein; instead, it typically leads to the silencing of the mutated gene due to the activation of a messenger RNA (mRNA) surveillance mechanism called nonsense-mediated mRNA decay (NMD) [1–4]. NMD specifically targets PTC-containing mRNAs for rapid degradation, which can be triggered either by the presence of at least one splicing event 50–55 nucleotides downstream of the PTC position or because of an abnormally long distance between the first stop codon encountered by the ribosome and the position of the poly(A) binding protein C1 (PABPC1) [5].

Some small molecules can induce the recruitment of a tRNA charged with an amino acid when the ribosome pauses at a PTC. This process, known as PTC readthrough [6, 7], allows translation to continue through a nonsense mutation within the original reading frame, producing a full-length protein that may differ from the wild type protein by a single amino acid, the residue incorporated when the ribosome reads the PTC. Among the translational readthrough-inducing drugs (TRIDs), certain members of the aminoglycoside family were identified as readthrough agents over 40 years ago [8]. ELX-02, a chemically engineered aminoglycoside reached the furthest stage in CF-specific clinical development, has so far only been tested in a small cohort of CF patients carrying the G542X variant, either alone or in combination with the CFTR potentiator ivacaftor. Phase 2 trial data revealed limited functional and clinical benefit, which may at least partially be explained by suboptimal pharmacodynamic properties, including low pulmonary drug concentrations [9, 10]. Despite their therapeutic potential in restoring gene expression, the development of aminoglycosides has been hindered due to the emergence of ototoxicity and nephrotoxicity following prolonged use [11, 12]. This toxicity has driven the search for alternative, non-aminoglycoside molecules with readthrough activity. More than twenty molecules have been reported to exhibit readthrough activity, some of which can also inhibit NMD, such as G418, amlexanox, cytochalasin D, jasplakinolide or Escin [13–16].

The mode of action of these molecules varies, including ribosome targeting, as observed with aminoglycosides, as well as ataluren [17, 18] to either favor the recruitment of a near-cognate tRNA or to prevent translation termination, respectively [19]. Some compounds, such as SRI-41315, SRI-37240, NVS1.1 or NVS2.1 accelerate the degradation of the translation termination complex (eRF1 or eRF3) [20, 21]. Others, like clitocine and FU-r, act through base substitution [22, 23], while certain molecules interfere with post-transcriptional tRNA modifications, as described for 2,6-diaminopurine (DAP), NV848, NV914 and NV930 compounds [24, 25]. Finally, the mode of action of some TRIDs remains to be elucidated, as is the case for RTC13 and RTC14 [26].

TRIDs are compounds with strong therapeutic potential for genetic diseases caused by a nonsense mutation. This is the case, for example in cystic fibrosis (CF), a genetic disease linked to a functional defect of the CFTR ion channel, in which nonsense mutations account for approximately 10% of cases [27]. To date, these patients do not have access to a curative treatment, unlike patients that hold other cystic fibrosis variants that are responsive to CFTR modulators, underlining the need to investigate TRIDs as a therapeutic strategy for CF as well as other genetic disorders [28]. However, attempts to bring TRIDs to patients have so far been unsuccessful. One possible reason is that the efficacy of these molecules in inducing translational readthrough must be high enough to provide a clinical benefit to patients.

A significant challenge in the field of TRIDS, is that all have been identified and characterized independently of one another in different cellular models across different labs and studies, making it difficult to assess their relative efficacy. Moreover, depending on the models used, a given molecule may display strong readthrough activity, or conversely, weak or even no activity at all. A well-documented example is ataluren, whose readthrough activity has been repeatedly debated [29–32]. Thus, ataluren has been shown to restore dystrophin expression in mdx mice carrying a UAA nonsense mutation in the *DMD* gene [33]. More recently, readthrough activity was clearly demonstrated in a retinal organoid model of AIPL1-associated Leber congenital amaurosis [29]. In contrast, ataluren was completely ineffective at restoring expression of the SCN5A sodium channel carrying a nonsense mutation in human induced pluripotent stem cell–derived cardiomyocytes [32]. Similarly, the readthrough activity of ataluren could not be confirmed in organoids derived from cells of patients with cystic fibrosis harboring a nonsense mutation [31]. Therefore, we set out to systematically conduct a comparative study of molecules with previously described readthrough activity, low toxicity, and commercial availability: Ataluren, CC-885, CC-90009, clitocine, DAP, ELX-02, Escin, FU-r, G418, SRI-37240, SRI-41315 and TLN468 [16, 21, 23, 25, 33–37]. We tested readthrough capacity in various cellular models ranging from transgenic reporter-based system, to primary patient-derived intestinal organoid models (PDIOs) with functional CFTR characterization using different assays. Finally, a standardized evaluation of cellular toxicity was performed using the same assays. This combined dataset can help in prioritization of the most effective compounds.

## Methods

### Cell culture

U2OS and Caov-3 cells were grown in Dulbecco’s modified Eagle’s medium with Glutamax (Gibco) containing 10% fetal calf serum (Sigma-Aldrich) and 1% zellshield (Minerva Biolabs). Caco-2 cells were grown in Minimum essential medium with Glutamax (Gibco) containing 20% of fetal calf serum (Sigma-Aldrich) and 1% zellshield (Minerva Biolabs). U20S cells were transfected with JetOptimus according to the supplier’s protocol (polyplus transfection).

Calu-6 cells were grown in Roswell Park Memorial Institute medium 1640 with Glutamax, containing 10% foetal calf serum (Sigma-Aldrich) and 1% zellshield (Minerva Biolabs).

The isogenic Fischer Rat Thyroid (FRT) cells (generously provided by the Cystic Fibrosis Foundation). were stably transduced using Flp-In^TM^ System (Thermo Fisher Scientific, Inc.) with either a human *CFTR* cDNA harboring the *CFTR* nonsense mutations (*Y122X, G542X, R553X*, and *W1282X*) under *CMV* promoter control. Cells were maintained at 37 °C and 5% CO_2_. When the culture reached 70–80% confluence, cells were recovered with 0.05% trypsin/EDTA(Gibco). At each passage cell viability was measured using Trypan blue (Sigma-Aldrich) exclusion test. After trypsin, cells were seeded on the Snapwell Costar insert 3407 (Corning) at 0.44x10^6^ cell/cm^2^ in Nutriment Mixture F-12 Ham (Sigma-Aldrich) complemented with hygromycin (Gibco) for 2 days. Then, cells were switch to air liquid interface in the same media without hygromycin and changed every 2 days. Experiments were performed 7 days after air-liquid interface.

Normal Human Dermal Fibroblast (NHDF) (Promocell) were grown in Basal Medium Eagle (Gibco) containing 10% foetal calf serum (Sigma-Aldrich), 2 mM L-glutamine (Thermo Fisher Scientific) and 1% zellshield (Minerva Biolabs).

### Firefly luciferase readthrough assay

Measurement of firefly luciferase readthrough assay has been described previously in detail [38].

### Forskolin induced swelling assay in PDIOs

Intestinal crypts were isolated from biopsies of subjects with cystic fibrosis as previously described [39]. Organoids were incubated in a humidified chamber with 5% CO2 at 37 °C. Medium was refreshed every 2–3 days, and organoids were passaged 1:4 every 7–8 days. Prior to forskolin induced swelling assay measurements, organoids were grown at least 3 weeks after crypt isolation or thawing. Functional assessment of CFTR function was assessed with the forskolin-induced swelling assay, performed as described by Vonk et al. [40]. All readthrough compounds were incubated for 48 hours. Organoid swelling was monitored during 180 minutes using a Zeiss LSM 710 confocal microscope and stimulated with 5 µM forskolin. Total organoid surface area per well was quantified based on calcein green staining as described by Vonk et al. The same cells used for CFTR functional characterization were used for viability analysis. After removal of the culture supernatant, fresh culture medium containing 10% Alamar Blue (Life Technologies) was added for 4 hours before measuring fluorescence using a Fluostar Optima (BMG Labtech).

### Short-circuit current (SSC) assay

SCC measurements were performed as previously described [41]. In brief, FRT cells seeded on inserts were mounted in an EM-CSYS-6 Ussing chamber system (Physiologic Instruments Inc., USA) made of two hemi-chambers, containing the following solutions: low Cl^-^ apical solution; 1.2 mM NaCl, 115 mM Na-gluconate, 25 mM NaHCO_3_, 1.2 mM MgCl_2_, 4 mM CaCl_2_, 2.4 mM KH_2_PO_4_, 1.24 mM K_2_HPO_4_, 10 mM mannitol (pH 7.4) and basolateral solution; 115 mM NaCl, 25 mM NaHCO_3_, 1.2 mM MgCl_2_, 1.2 mM CaCl_2_, 2.4 mM KH_2_PO_4_, 1.24 mM K_2_HPO_4_, 10 mM glucose (pH 7.4). All readthrough compounds were incubated for 48 hours.

### Late-stage toxicity assay

The toxicity assay was performed 24 h after starting cell treatment in 2 ml cell culture (6-cm dish). The cell culture supernatant was centrifuged at 6000×g for 5 min to pellet the cells and cell debris. Supernatant was then used to measure adenylate kinase activity according to the supplier’s protocol (Lonza Biosciences). Luciferase activity was then measured with a Tristar luminometer (Berthold).

### Early-stage toxicity assay

The same cells used for the late-stage toxicity assay were incubated, after removal of the culture supernatant, with fresh culture medium containing 10% Alamar Blue (Life Technologies) for 4 hours. Fluorescence was then measured using a Fluostar Optima (BMG Labtech).

### Western-Blot assay

A total of 2.5 × 10^5^ cells was lysed, and proteins were resolved by 10% SDS–PAGE before transfer onto nitrocellulose membranes. Membranes were incubated overnight at 4 °C with either anti-p53 antibody (DO1, 1:200; Santa Cruz Biotechnology) or anti-IMPORTIN9 antibody (1:1000; Abcam). Following three washes in TBS-T, membranes were probed with HRP-conjugated secondary antibodies specific for mouse (1:5000; Jackson ImmunoResearch) or rabbit (1:50,000)) primary antibodies. Signals were detected using SuperSignal West Femto Maximum Sensitivity Substrate (Pierce Biotechnology, Rockford, IL, USA).

## Results

### Comparative readthrough efficiency of TRIDs on the three stop codons and on spliced or unspliced mRNAs

To identify TRIDs, screening systems rely either on a construct carrying one or two cDNAs with a PTC or on a construct containing a cDNA interrupted by an intron located downstream of a PTC. The first type of construct allows the synthesis of mRNAs carrying a PTC that are not subjected to NMD in human cells [33, 42, 43]. The second type of construct generates mRNAs carrying a PTC that are subjected to NMD due to a splicing event downstream of the PTC position [21, 38]. Therefore, it is possible that a readthrough molecule may be effective in correcting a PTC on an unspliced mRNA but may show little or no activity on a PTC located on a spliced mRNA, or vice versa.

**Fig. 1:**
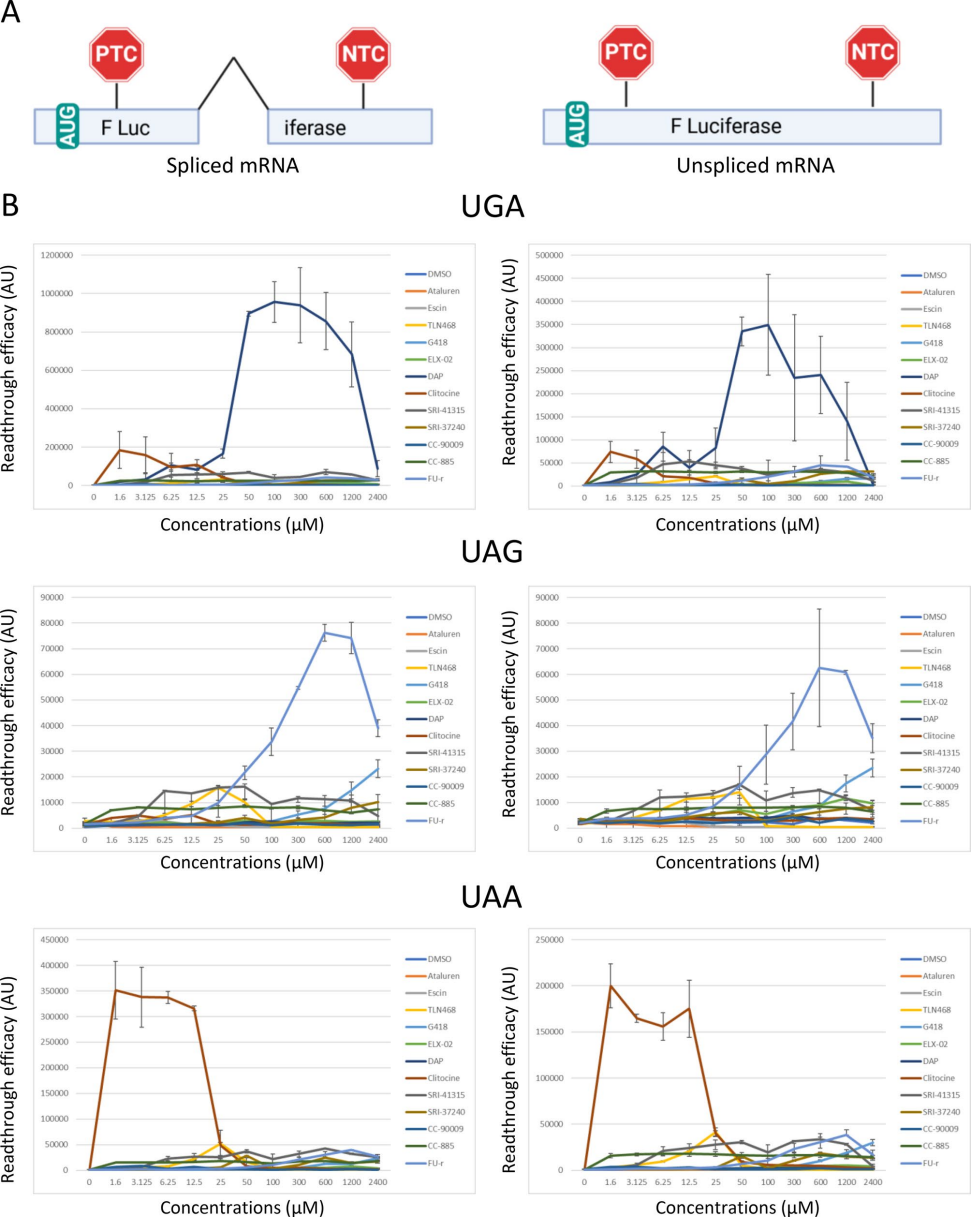
Comparative analysis of translational readthrough efficiencies of different TRIDs using an exogenous luciferase reporter system. (**A**) Schematic representation of the mRNA derived from two luciferase constructs used. On the left, the mRNA is transcribed from a construct that consists of the cDNA encoding firefly luciferase interrupted by an intron and an upstream premature termination codon (PTC). On the right, the mRNA is transcribed from a construct that consists of a cDNA encoding firefly luciferase interrupted by a premature termination codon (PTC). The translation initiation codon is indicated (AUG). The physiological stop codon is indicated as NTC (natural termination codon). (**B**) U2OS cells were transfected with the construct containing an intron or with the intronless construct before being incubated with increasing doses of TRIDs, followed by measurement of luciferase activity after 24 hours. The identity of the PTC is indicated above each histogram (UGA at the top, UAG for the two middle histograms, and UAA for the bottom histograms). The results are representative of three independent experiments

To address this, we previously designed an intron-free version of the constructs expressing an mRNA encoding firefly luciferase, with an intron disrupting the reading frame and a PTC located more than a hundred nucleotides upstream of the intron [38] (Fig. 1A). U2OS cells were transfected with these different constructs and then incubated with increasing doses of various previously reported TRIDs (Fig. 1B). As previously reported, readthrough efficiency depends on both the identity of the stop codon and the molecule used. For the UGA PTC, DAP stands out in terms of efficiency compared to other molecules tested in this cell model. Two other compounds, clitocine and SRI-41315, show lower efficiency than DAP but higher than the other tested molecules. For the UAG PTC, FU-r is the most effective molecule for correcting this PTC, followed by G418, then TLN468 and SRI-41315. Finally, for the UAA PTC, clitocine exhibits the highest correction efficiency, followed by TLN468 and SRI-41315. Interestingly, the ranking remained consistent regardless of whether readthrough efficiency was assessed on spliced or unspliced mRNA (Fig. 1 left and right column). From this experiment, the three most effective molecules for each stop codon, along with their optimal concentrations, defined as the lowest concentration yielding maximal activity, were identified (Table 1). Of note, the most effective TRID differed among the three stop codons, with DAP for UGA, FU-r for UAG, and clitocine for UAA. Only one compound, SRI41315, appears among the three most effective molecules for all three types of stop codons. Clitocine and TLN468 were consistently found among the three most effective molecules for two stop codons: UGA and UAA, or UAG and UAA, respectively. Finally, while certain molecules such as DAP or FU-r required relatively high concentrations (100 and 600 µM, respectively) to achieve maximal readthrough activity, others, including clitocine and SRI-41315, were effective at lower concentrations (1.6 and 6.25 µM, respectively).

**Table 1:** Summary of the results obtained in Fig. 1 for the three best TRIDs for each PTC

	Rank	TRID	Concentration (μM)
UGA	1	DAP	100
	2	Clitocine	1.6
	3	SRI-41315	6.25
UAG	1	FU-r	600
	2	SRI-41315	6.25
	3	TLN468	25
UAA	1	Clitocine	1.6
	2	TLN468	25
	3	SRI-41315	6.25

### Readthrough efficiency of TRIDs on endogenous nonsense mutations

**Fig. 2:**
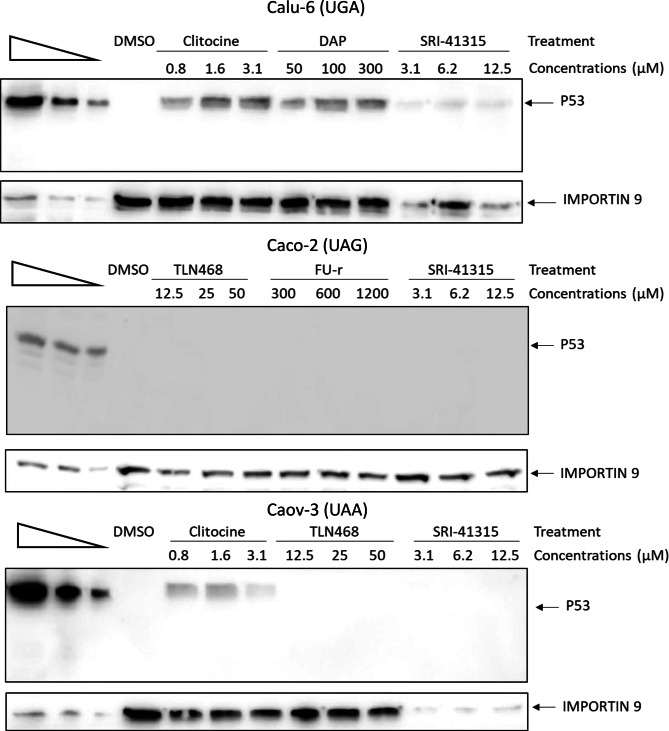
Readthrough efficiency on endogenous nonsense mutations. Western blot analysis of protein extracts from calu-6 cells (upper panel; UGA nonsense mutation), caco-2 cells (middle panel; UAG nonsense mutation), or caov-3 cells (bottom panel; UAA nonsense mutation). The three lanes on the left correspond to serial dilutions of a protein extract from U2OS cells carrying a wild-type TP53 gene. Importin-9 was used as a loading control

We next characterized the most effective TRIDs on endogenous nonsense mutations to validate the results obtained with the firefly luciferase reporter system. Calu-6 cells harboring a UGA nonsense mutation in the *TP53* gene, Caco-2 cells carrying a UAG nonsense mutation in *TP53*, and Caov-3 cells with a UAA nonsense mutation in *TP53* were incubated for 24 hours with one of the three most effective TRIDs identified in Fig. 1. Specifically, DAP, clitocine, and SRI-41315 were tested on the UGA mutation (Fig. 2A); FU-r, SRI-41315, and TLN468 were assessed on the UAG mutation (Fig. 2B); and clitocine, SRI-41315, and TLN468 were evaluated on the UAA mutation (Fig. 2C). Each compound was applied at three concentrations surrounding the optimal values indicated in Table 1. Readthrough efficiency on a protein level was subsequently analyzed by Western blotting (Fig. 2).

As expected, in the absence of treatment (DMSO), P53 protein was undetectable in all three cell lines due to the presence of a PTC in *TP53*. In Calu-6 cells (UGA mutation), *TP53* expression was restored by treatment with clitocine or DAP. By contrast, SRI-41315 consistently caused a pronounced reduction in overall protein abundance, as indicated by the marked decrease in IMPORTIN 9 levels, which served as the loading control. Consequently, no detectable P53 synthesis was observed under these conditions with SRI-41315.

In Caco-2 cells (UAG mutation), none of the tested TRIDs restored detectable P53 expression. Interestingly, in this cell line, SRI-41315 did not have a pronounced effect on global protein expression, since IMPORTIN 9 levels appeared unaffected by the compound.

Finally, in Caov-3 cells (UAA mutation), only clitocine successfully restored P53 synthesis. Again, treatment with SRI-41315 marked a strong reduction in the overall amount of extracted proteins, consistent with the effect observed in Calu-6 cells.

Taken together, these results demonstrate that, in the tested cellular models harboring endogenous nonsense mutations in *TP53* and assessed by Western blot analysis, only clitocine and DAP were capable of restoring P53 expression, specifically in the context of UGA and/or UAA stop codons.

### Evaluation of the toxicity of various TRIDs

A key consideration in the development of TRIDs with therapeutic potential is their safety profile. Toxicity can be assessed using various approaches, but it is typically categorized into early or late toxicity evaluations. To maintain consistency with previously published studies, toxicity was measured after 24 hours of treatment.

**Fig. 3:**
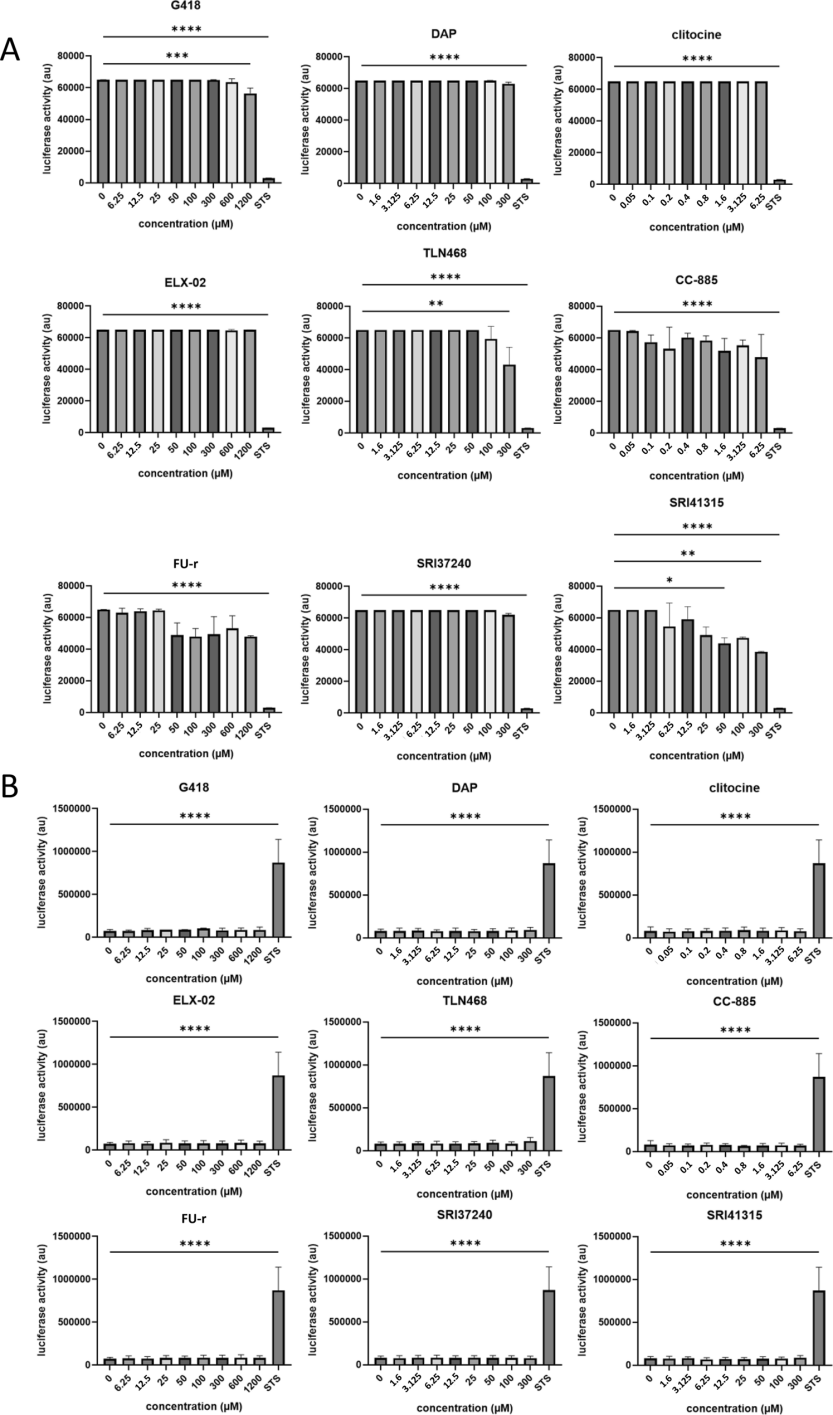
Assessment of the toxicity of the different TRIDs as a function of concentration. (**A**) Early toxicity measurement using alamar blue staining. (**B**) Late toxicity measurement based on adenylate kinase activity in the cell culture medium. Data were obtained from three independent experiments. *p*-values were calculated by T-test *<0.05; **<0.01; ***<0.001; **<0.0001

Toxicity assessments were performed on normal primary human fibroblast cells, as established immortalized-cell lines tend to exhibit higher resistance to cell death compared to primary cells. Two assays were conducted on the same samples: an early toxicity assessment based on mitochondrial activity using Alamar Blue staining (Fig. 3A), and a late toxicity evaluation by measuring adenylate kinase activity in the cell culture supernatant (Fig. 3B).

A wide range of concentrations around the optimal concentration indicated in Table 1 was used for each TRID. As shown in Fig. 3A, apart from treatment with staurosporine, a pro-apoptotic agent used as a positive control, SRI41315 and at lesser extend G418 and TLN468 induce significant toxicity starting at 50, 1200 or 300 µM, respectively. The other TRIDs do not induce any detectable early toxicity at all tested concentrations and after 24 hours of treatment.

Interestingly, late toxicity analysis, as measured by adenylate kinase activity in the culture medium, revealed no detectable toxicity for any of the TRIDs except following staurosporine treatment (Fig. 3B). Altogether, the results presented in Fig. 3 indicate that, under the tested experimental conditions, the TRIDs do not induce any early or late toxicity, with the exception of SRI-41315, G418, and TLN468.

### Comparative analysis of functional CFTR restoration in PDIOs from individuals with cystic fibrosis

**Fig. 4:**
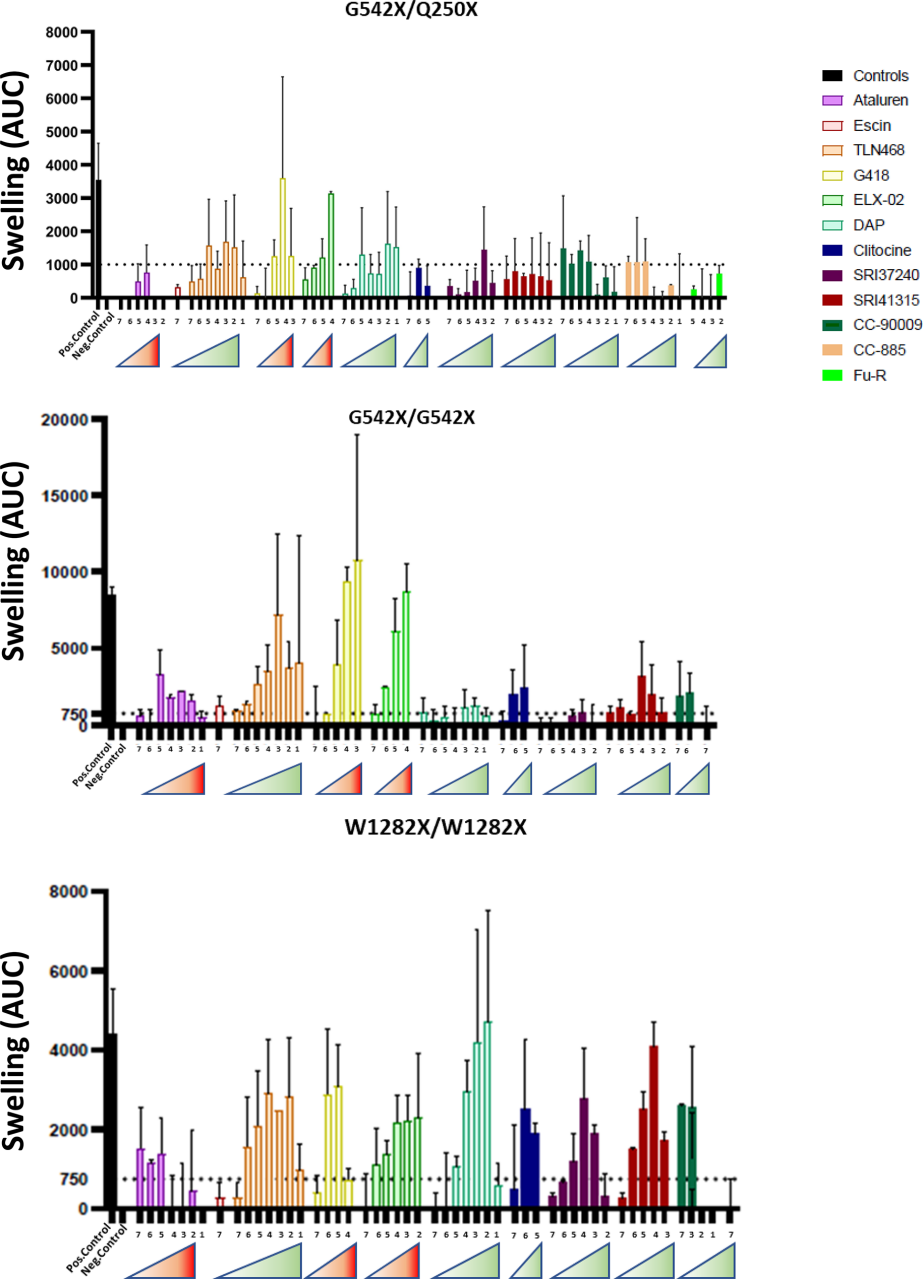
Comparison of CFTR function restoration efficiency by FIS assay in PDIOs derived from people with cystic fibrosis carrying the nonsense mutations G542X/Q250X (top panel), G542X/G542X (middle panel), or W1282X/W1282X (bottom panel). PDIOs were incubated with increasing doses of TRIDs before measuring organoid swelling. The concentrations used for molecules marked with a red-gradient triangle were 10, 50, 100, 300, 600, 1200, and 2400 µM, and for those marked with a green-gradient triangle: 0.1, 0.5, 1, 10, 25, 50, and 100 µM. Results represent the mean of two independent experiments with three technical replicates per experiment

To evaluate the ability of TRIDs to restore CFTR expression in an isogenic context, we used patient-derived intestinal organoids (PDIOs) carrying either the heterozygous nonsense mutations G542X (UGA)/Q250X (UAG or UAA), the homozygous G542X (UGA) variant, or the homozygous W1282X (UGA) variant (Fig. 4).

PDIOs are three-dimensional cellular models, self-organizing into structures that recapitulate key structural and functional features of native tissues. They therefore provide a preclinical system that may reduce the need for animal models, specifically in the context of CF due to the previous development of CFTR-dependent functional assays [44–46]. Particularly, CFTR channel function can be quantitatively assessed using the forskolin-induced swelling (FIS) assay in which forskolin stimulation triggers CFTR-mediated ion transport and subsequent water transport into the PDIO lumen, resulting in a measurable increase in PDIO size [40].

We compared FIS in PDIOs harboring three different genotypes: G542X/Q250X (UGA/UAA), G542X/G542X (UGA/UGA) and W1282X (UGA/UGA) across a concentration range of all TRIDs. Based on the experiments above, we divided the TRIDs in whether they needed a higher or lower concentration range to be effective (red: higher concentrations, green: lower concentrations). After the FIS assay we immediately characterized viability with Alamar Blue in the same cells, allowing exclusion of data when viability was significantly hampered (<65%). As positive control for CFTR function, we treated the cells with a combination of ELX-02, NMD inhibitor SMG1i and CFTR modulators elexacaftor/tezacaftor/ivacaftor (ETI), which we previously described as very effective [39]. In PDIOs harboring G542X/Q250X mutations, all TRIDs except Escin achieved, at least at one tested concentration, a substantial level of CFTR functional restoration, as indicated by the dotted threshold line at a 1000 AUC, which based on previous experiments is regarded as noise-limit. The TRIDs producing swelling amplitudes closest to the control condition were G418, ELX-02, TLN468, and, with lower efficacy, DAP, CC-90009, and SRI-41315.

In homozygous G542X PDIOs, most TRIDs tested induced substantial CFTR functional recovery at one or more concentrations. The most effective compounds were G418, ELX-02, and TLN468.

Finally, in PDIOs homozygous for W1282X, all TRIDs except Escin substantially restored CFTR function. Among these, DAP, SRI-41315, and G418 emerged as the most effective compounds.

Together, these results demonstrate that several TRIDs are able to restore CFTR function in PDIO models carrying different nonsense mutations, although the efficacy of individual compounds varies depending on the mutation context.

### Evaluation of CFTR functional restoration by short-circuit current measurements

**Fig. 5:**
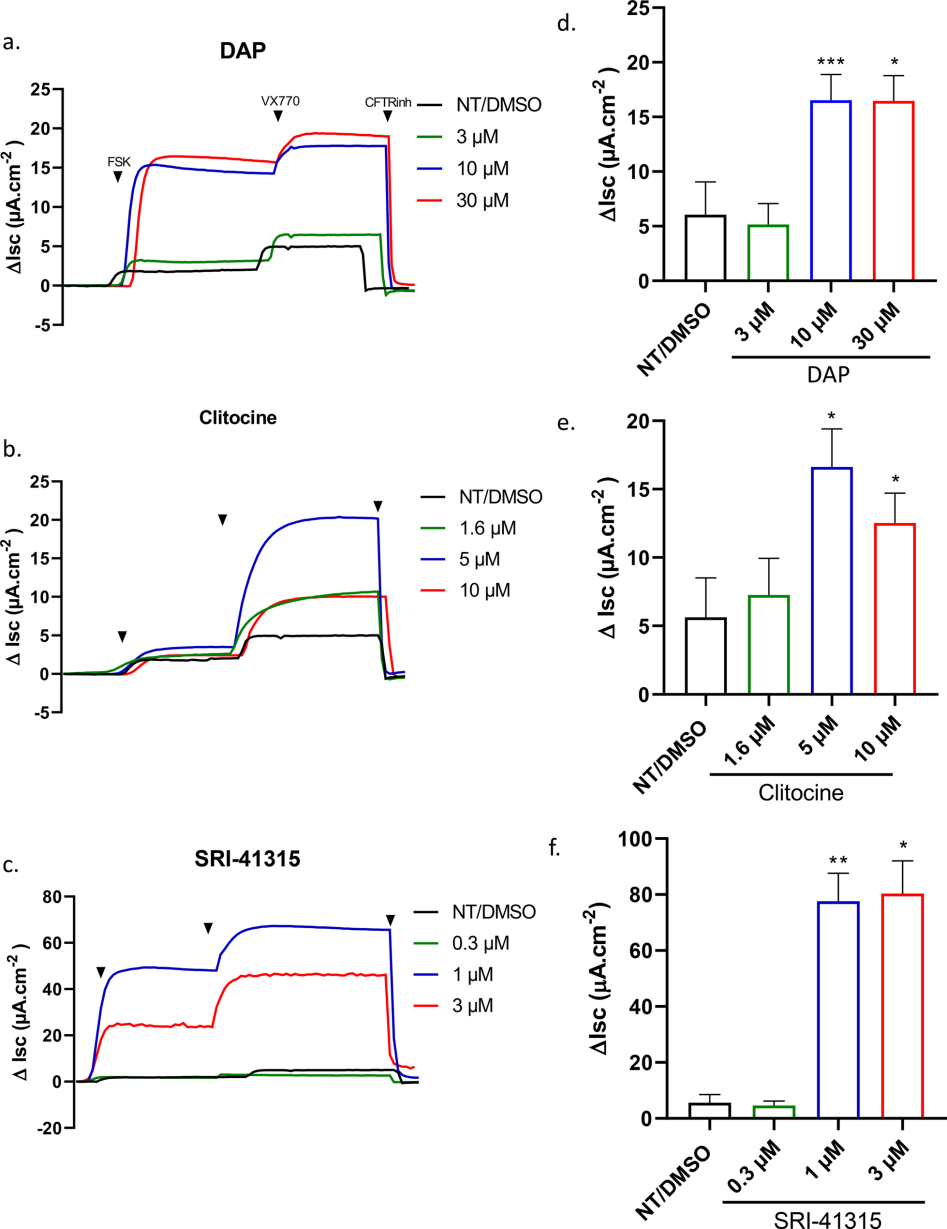
Rescue of CFTR-W1282X short-circuit currents (isc) in fisher rat thyroid (FRT) cells by TRIDs. Original tracings of isc as function of time for CFTR-W1282X cells incubated 48 h with **a**) DAP; **b**) Clitocine; and **c**) SRI-41315. CFTR-W1282X isc was stimulated by 1 μM forskolin (FSK) and then 1 μM V × 770 and finally inhibited by 10 μM CFTRinh172. **d**- **f**) Mean ±sd of isc FSK and V × 770 for FRT cells treated with d. DAP, **e**. Clitocine and **f**. SRI-41315 and statistically analyzed with paired t test.*: *p* < 0.05 **: *p* < 0.01, ***: *p* < 0.001

To further characterize the three compounds that showed the highest activity across multiple models (DAP, clitocine and SRI-41315), they were assessed for their ability to restore CFTR function in Fisher Rat Thyroid (FRT) cells engineered to express a non-spliced human CFTR mRNA harboring homozygous nonsense mutations (W1282X, R553X, G542X, or Y122X). CFTR channel function was evaluated by culturing cells at the air–liquid interface and measuring transepithelial short-circuit current in Ussing chambers following forskolin stimulation. The results presented in Fig. 5 and Supplemental Figs. 1–3 demonstrate that all three compounds elicited a forskolin-dependent transepithelial current that was further enhanced by the addition of the potentiator VX-770 in cells carrying the W1282X (Fig. 5), G542X (Supplemental Fig. 1), and R553X (Supplemental Fig. 2) mutations. In contrast, in cells harboring the Y122X mutation (UAA), only DAP failed to induce a measurable current, indicating a lack of CFTR functional restoration (Supplemental Fig. 3). This observation is consistent with the mutation type, as Y122X corresponds to a UAA rather than a UGA nonsense mutation for W1282X, R553X and G542X. It is noteworthy that, in this experimental model, TRID concentrations were adjusted to prevent cytotoxicity associated with prolonged exposure. Accordingly, DAP and SRI-41315 were applied at suboptimal concentrations, whereas Clitocine was tested at higher concentrations to allow detection of more pronounced effects. SRI-41315 appears to be the most effective TRID to rescue CFTR function in this model and under these experimental conditions.

Overall, these results confirm that TRIDs can restore CFTR channel activity in several nonsense mutation contexts, although their efficacy depends both on the identity of the stop codon and on compound-specific dosing constraints.

## Discussion

In this comparative study of multiple TRIDs, four distinct cellular models were employed, which revealed a degree of variability in their responses. The exogenous functional assay using a firefly luciferase reporter construct provided an initial evaluation of 12 TRIDs across the three stop codons (Fig. 1). From this analysis, some compounds clearly stood out, such as DAP for UGA codons, clitocine for UAA codons, and to a lesser extent FU-r for UAG codons, while the remaining TRIDs displayed only modest and comparable readthrough activity. Notably, ataluren and Escin showed no detectable readthrough activity in this assay.

Readthrough activity observed in the luciferase reporter system was subsequently validated in an endogenous nonsense mutation model (Fig. 2), where DAP and clitocine restored protein expression, whereas the other tested compounds did not (TLN468, FU-r and SRI-41315). Importantly, this Western blot analysis is not a functional assay per se, as it only assesses restoration of protein synthesis without addressing whether the rescued protein is functional. In this context, only the TRIDs that exhibited the strongest luciferase activity were able to restore detectable P53 expression, thus confirming correction of an endogenous nonsense mutation. Interestingly, SRI-41315 profoundly reduced overall protein expression in two cell lines. This effect could be related with its mechanism of action, which interferes with translation termination to promote readthrough. One possible explanation is that Calu-6 and Caov-3 cells may be particularly sensitive to impaired translation termination.

Cytotoxicity profiling of the TRIDs under the experimental conditions employed (specifically 24 hours of incubation) showed that only G418, SRI-41315, and TLN468 exhibited measurable toxicity. However, the observed effects were substantially milder than those induced by staurosporine, used as a positive control (Fig. 3). This finding suggests that activation of readthrough does not inherently cause acute deleterious effects on cell viability, at least in the short term. This observation might be particularly relevant when considering TRIDs as potential therapeutic candidates.

It is also noteworthy that early (Alamar Blue assay) and late (adenylate kinase release assay) toxicity measurements did not yield consistent results: while the former detected toxicity for a subset of TRIDs, the latter indicated no significant cytotoxicity. Microscopic inspection did not reveal substantial detachment or accumulation of floating cells but showed a trend toward a more rounded morphology following treatment with G418, TLN468, and SRI-41315 (data not shown). These observations further support the notion that the TRIDs tested do not induce massive or rapid toxicity. Nonetheless, early-stage toxicity assays appear more suitable for detecting subtle effects and therefore may be more informative for assessing potential safety risks.

Functional assessment of CFTR activity using the forskolin-induced swelling (FIS) assay in patient-derived intestinal organoids carrying nonsense CFTR mutations provided additional insights (Fig. 4). Across different genotypes, a variable degree of functional rescue was observed, with some TRIDs effective in certain contexts but not others. G418, TLN468, and ELX-02 restored CFTR function across all three genotypes tested. This is a particularly interesting finding, as neither G418 nor ELX-02 appeared among the most active compounds in the luciferase assay (Fig. 1), likely reflecting differences in the amino acids inserted at PTCs. DAP, clitocine, and SRI-41315 were also able to restore CFTR channel activity, although not more effectively than other TRIDs in this model.

Further evaluation of CFTR function by short-circuit current measurements in Ussing chambers, using FRT cells expressing homozygous CFTR nonsense variants, demonstrated that DAP, clitocine, and SRI-41315 were all capable of inducing functional rescue (Fig. 5 and Supplemental Figs. 1–3). In this system, however, SRI-41315 was the most effective compound across all four tested mutations.

Taken together, three types of functional assays were employed in this study. It is important to emphasize that a functional assay does not directly reflect the intrinsic readthrough efficiency of a TRID. A compound may induce robust readthrough, enabling efficient translation of a nonsense-containing mRNA, but if the amino acid incorporated at the PTC differs from the wild-type residue, the resulting protein may lack functionality. Conversely, functional rescue may be observed even in cases of modest readthrough. Thus, functional assays strictly evaluate restoration of protein activity, and outcomes may vary considerably for the same TRID depending on the mutation tested. It is therefore critical to assess candidate TRIDs across multiple functional assays and, ideally, within the patient-specific mutational context.

Additional factors beyond the identity of the inserted amino acid must also be considered. For instance, DAP emerged as the most effective compound for restoring activity of UGA-containing firefly luciferase (Fig. 1) and was among the most effective in rescuing CFTR function in W1282X organoids using the FIS assay (Fig. 4), on par with SRI-41315. However, in Ussing chamber measurements of the same W1282X mutation, SRI-41315 was markedly more effective (Fig. 5). Similarly, while clitocine appeared less effective than DAP or SRI-41315 in the FIS assay, it was comparably effective in the Ussing chamber assay. These discrepancies may reflect experimental conditions: for example, DAP was applied at suboptimal concentrations in the Ussing chamber assay to avoid long-term toxicity. Differences in cell type, compound permeability, and culture conditions (e.g., salt content influencing solubility) may also contribute.

Overall, this study identifies DAP, SRI-41315, clitocine, and TLN468 as the most consistently effective TRIDs across multiple experimental systems. These results align with a recently published large-scale comparative study evaluating readthrough efficiency on ~ 5800 PTCs using an NGS–fluorescence approach, which identified DAP and clitocine as the most effective TRIDs for UGA and UAA codons, respectively, while SRI-41315 was reported as less effective and TLN468 was not tested [47]. Another comparative study on a smaller scale showed, in the context of hemophilia, that DAP is the most effective TRID for most of the nonsense mutations tested, compared with G418 or ELX-02 [48]. Despite these advances, it remains challenging to define a single assay, or even a set of assays, that could reliably predict clinical efficacy. To date, only two TRIDs, ataluren and ELX-02, have reached clinical trials, and neither demonstrated sufficient therapeutic benefit due to limited efficacy. No clear threshold of activity has been established to guide preclinical and clinical development decisions. At present, the minimal benchmark is set by ataluren and ELX-02, and any new TRID must demonstrate efficacy well above these levels to warrant clinical evaluation. Ultimately, candidate TRIDs must be assessed using multiple assays that closely replicate the patient-specific genetic context to maximize the likelihood of therapeutic success. This is all the truer as readthrough efficiency has already been shown to be influenced by both the identity of the stop codon and the surrounding nucleotide context [43, 49–52]. Finally, it is very difficult to predict whether a TRID will be effective in patients, since only two molecules have reached clinical trials, without enabling the identification of the key parameters required for successful correction of nonsense mutations by a TRID. In vivo evaluation of TRIDs therefore represents an essential step prior to considering clinical trials, as it allows the establishment of pharmacokinetic and biodistribution profiles, as well as the assessment of the efficacy of nonsense mutation correction within a whole organism.

## Conclusion

The study presented here underscores the marked variability in TRID activity across experimental systems and nonsense mutation contexts. Although reporter assays provide a convenient initial estimation of readthrough efficiency, they do not necessarily predict the restoration of endogenous protein expression or functional activity. Our findings indicate that TRID efficacy is strongly influenced by the cellular model, the specific premature termination codon, and the functional requirements of the rescued protein. Among the compounds tested, DAP, SRI-41315, clitocine, and TLN468 showed the most consistent activity across several experimental settings, although their relative performance differed depending on the assay employed as summarized in Supplemental Table 1.

Taken together, these results highlight the importance of evaluating candidate TRIDs using multiple complementary models, including systems that closely reflect patient biology. In view of the limited clinical success achieved so far with readthrough-based therapies, additional preclinical investigations, particularly in vivo studies, will be necessary to better define the determinants of therapeutic efficacy and to support the development of more effective treatments for genetic diseases caused by nonsense mutations.

## Electronic supplementary material

Below is the link to the electronic supplementary material.

Supplementary Material 1

Supplementary Material 2

## Data Availability

The authors state that all data and materials used in this study are available upon request and subject to MTA conditions.

## Abbreviations

AUC: Area under the curve
cDNA: Complementary desoxynucleotide acid
CF: Cystic fibrosis
CFTR: Cystic fibrosis transmembrane conductance regulator
DAP: 2,6-diaminopurine
DMSO: Dimethyl sulfoxide
eRF: Eukaryotic release factor
ETI: Elexacaftor/tezacaftor/ivacaftor
FRT: Fischer rat thyroid
NMD: Nonsense-mediated mRNA decay
PABPC1: Poly(A) binding protein cytoplasmic 1
PDIO: Patient-derived intestinal organoid
PTC: Premature termination codon
TRID: Translational readthrough inducing drug
