# Supplementary material for: Comparative evaluation of TRIDs : a strategy to improve treatments
Source: J Transl Med. 2026 Mar 31;24:661. doi: 10.1186/s12967-026-08058-5 (PMC13162490; doi:10.1186/s12967-026-08058-5)
Supplement: Supplementary file 1 — Supplementary Material 1 [file 12967_2026_8058_MOESM1_ESM.pdf]

Model	Figure	sequence	Clitocine	DAP	SRI-41315	TLN468
Firefly luciferase	1 - UGA	**GAACGACATTTGAAATGAACGTG**	++	++++	+	-
	1 - UAG	**GAACGACATTTAGAATGAACGTG**	+	-	++	++
	1 - UAA	**GAACGACATTTAAAATGAACGTG**	++++	-	++	++
TP53	2 - Calu-6	**GCATCTTATCTGAGTGGAAGGAA**	++++	++++	+	NT
	2 - Caco-2	**TTTGCGTGTGTAGTATTTGGATG**	NT	NT	-	-
	2 - Caov-3	**GATGTTTTGCTAACTGGCCAAGA**	++++	NT	-	-
CFTR	4 - G542X / Q250X	**TATAGTTCTTTGAGAAGGTGGAA**<br>**GTACAGAGATTAGAGAGCTGGGA**	-	++	-	++
	4 - G542X	**TATAGTTCTTTGAGAAGGTGGAA**	+	-	-	+++
	4 - W1282X	**TTTGCAACAGTGAAGGAAAGCCT**	++	++++	+++	++
	5 - W1282X	**TTTGCAACAGTGAAGGAAAGCCT**	++	++	++++	NT
	Sup. Fig1 - G542X	**TATAGTTCTTTGAGAAGGTGGAA**	++	++	++++	NT
	Sup. Fig2 - R553X	**TGGAGGTCAATGAGCAAGAATTT**	++	+	+++	NT
	Sup Fig3 - Y122X	**TATCGCGATTTAACTAGGCATAG**	++	-	++++	NT

Supplemental Table 1: Comparative summary of the efficacy of clitocine, DAP, SRI-41315, and TLN468 across all figures of the study. The number of "+" signs indicates the degree of efficacy, with ++++ indicating the highest efficacy; + indicating low activity; − indicating no activity; and NT indicating not tested.
