# Supplementary material for: Comparative evaluation of TRIDs : a strategy to improve treatments
Source: J Transl Med. 2026 Mar 31;24:661. doi: 10.1186/s12967-026-08058-5 (PMC13162490; doi:10.1186/s12967-026-08058-5)
Supplement: Supplementary file 2 — Supplementary Material 2 [file 12967_2026_8058_MOESM2_ESM.pdf]

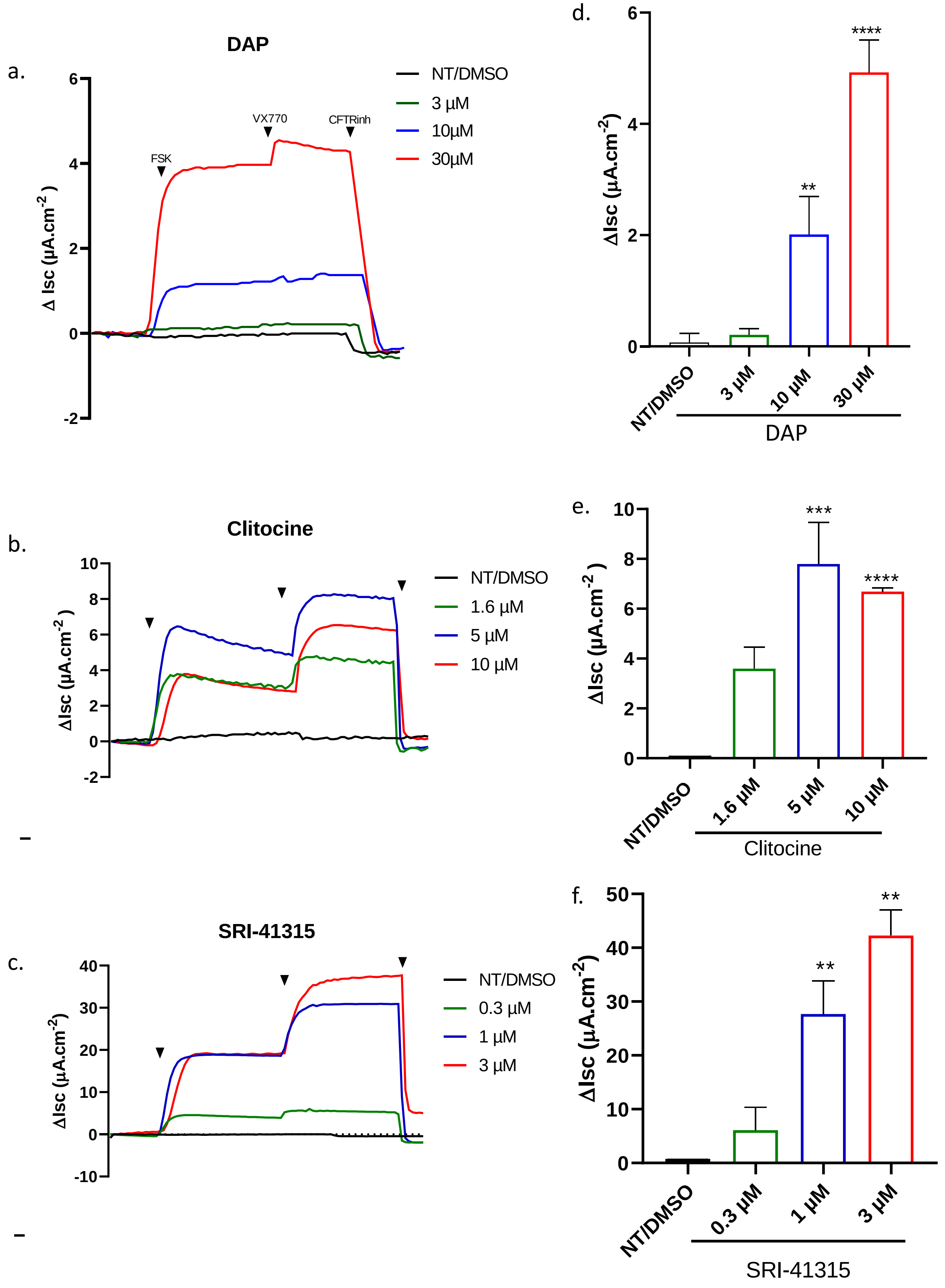

Supplemental Fig 1: **Rescue of CFTR-G542X short-circuit currents (Isc) in Fisher Rat Thyroid (FRT) cells by readthrough molecules**. Original tracings of Isc as function of time for CFTR-G542X cells incubated 48 h with a)2,6-Diaminopurine (DAP); b) Clitocine; and c) SRI-41315. CFTR-G542X Isc was stimulated by forskolin (FSK) at 1 µM (FSK) and then 1 µM VX770 and finally inhibited by 10 µM CFTRinh172. d- f) Mean±SD of Isc FSK and VX770 for FRT cells treated with d. 2,6-Diaminopurine, e. Clitocine and f. SRI41315 and statistically analysed with paired t test. **: p<0.01, ***: p<0.001, ****: p<0.0001.

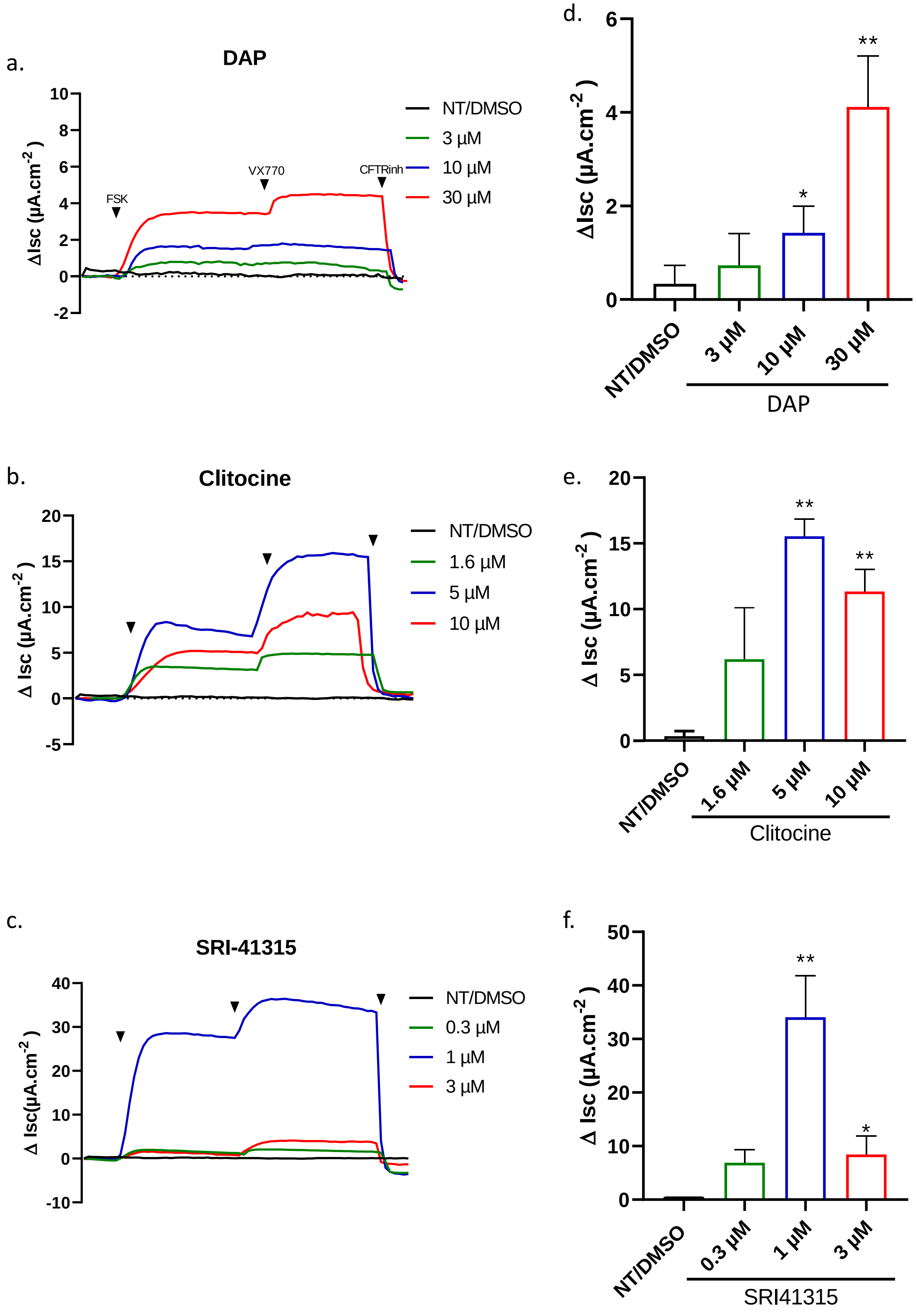

Supplemental Fig 2: **Rescue of CFTR-R553X short-circuit currents (Isc) in Fisher Rat Thyroid (FRT) cells by readthrough molecules**. Original tracings of Isc as function of time for CFTR-R553X cells incubated 48 h with a) 2,6-Diaminopurine (DAP); b) Clitocine; and c) SRI-41315. CFTR-R553X Isc was stimulated by forskolin (FSK) at 1 µM (FSK) and then 1 µM VX770 and finally inhibited by 10 µM CFTRinh172. d- f) Mean±SD of Isc FSK and VX770 for FRT cells treated with d. 2,6-Diaminopurine, e. Clitocine and f. SRI41315 and statistically analysed with paired t test. *: p<0.05 **: p<0.01.

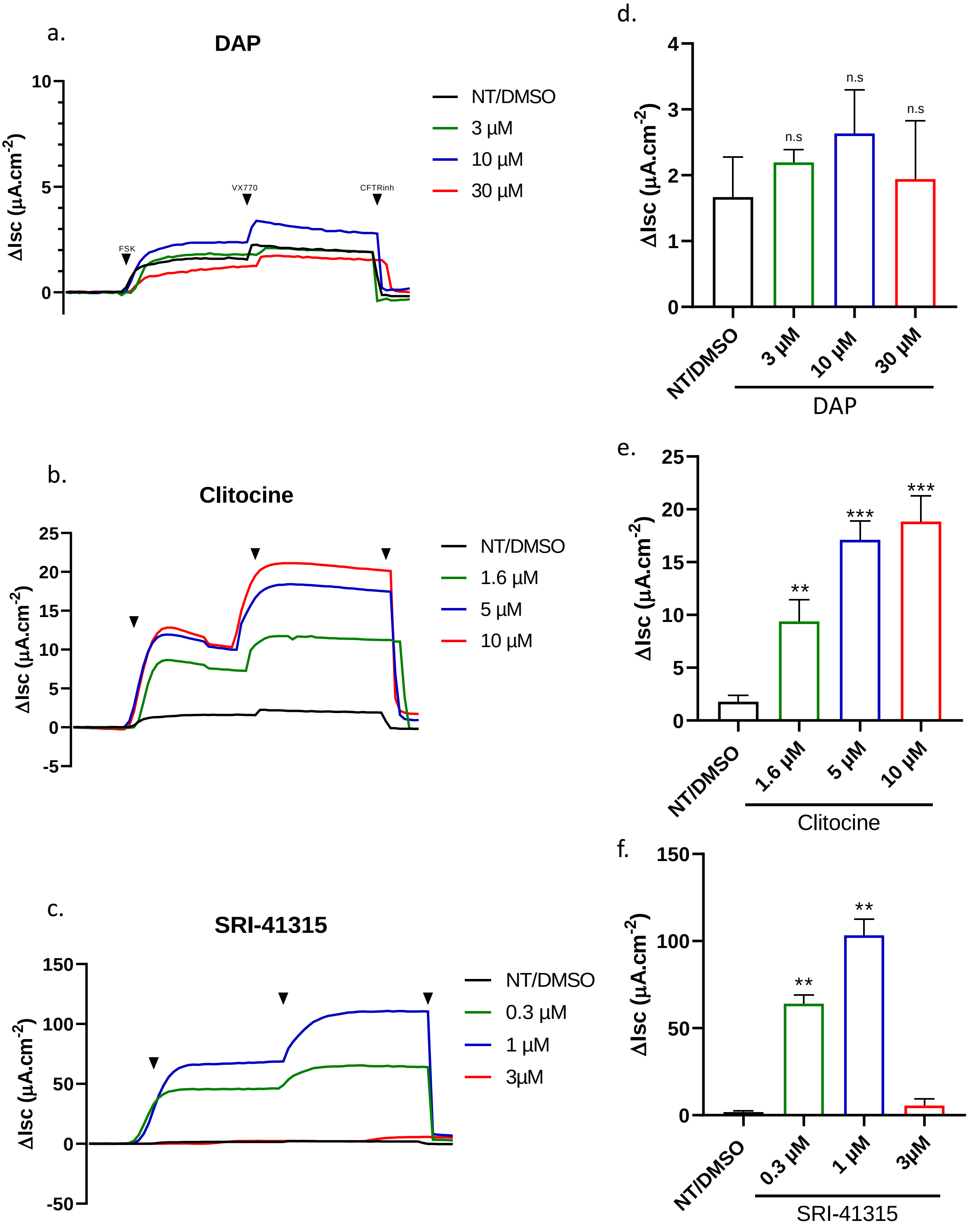

Supplemental Fig 3: **Rescue of CFTR-Y122X short-circuit currents (Isc) in Fisher Rat Thyroid (FRT) cells by readthrough molecules**. Original tracings of Isc as function of time for CFTR-Y122X cells incubated 48 h with a)2,6-Diaminopurine (DAP); b) Clitocine; and c) SRI-41315. CFTR-Y122X Isc was stimulated by forskolin (FSK) at 1 µM (FSK) and then 1 µM VX770 and finally inhibited by 10 µM CFTRinh172. d- f) Mean±SD of Isc FSK and VX770 for FRT cells treated with d. 2,6-Diaminopurine, e. Clitocine and f. SRI41315 and statistically analysed with paired t test . **: p<0.01, ***: p<0.001, n.s: not significant.
